# Comparison of friction produced by two types of orthodontic bracket protectors

**DOI:** 10.1590/2176-9451.19.1.086-091.oar

**Published:** 2014

**Authors:** Steyner de Lima Mendonça, Otávio José Praxedes Neto, Patricia Teixeira de Oliveira, Patricia Bittencourt Dutra dos Santos, Fábio Henrique de Sá Leitão Pinheiro

**Affiliations:** 1 MSc in Orthodontics, Potiguar University (UNP).; 2 PhD in Health Sciences, Federal University of Rio Grande do Norte (UFRN). Associate professor, UNP.; 3 PhD in Clinical Stomatology, Catholic University of Rio Grande do Sul (PUC-RS). Associate professor, UNP.; 4 Assistant professor, State University of Rio Grande do Norte (UERN).; 5 PhD in Orthodontics, University of Manchester. Associate professor, UNP.

**Keywords:** Orthodontics, Orthodontic brackets, Friction

## Abstract

**Introduction:**

Fixed orthodontic appliances have been regarded as a common causative factor of oral lesions. To manage soft tissue discomfort, most orthodontists recommend using a small amount of utility wax over the brackets in order to alleviate trauma. This *in vitro* study aimed at evaluating friction generated by two types of bracket protectors (customized acetate protector [CAP] and temporary resin protector [TRP]) during the initial stages of orthodontic treatment.

**Methods:**

An experimental model (test unit) was used to assess friction. In order to measure the friction produced in each test, the model was attached to a mechanical testing machine which simulated maxillary canines alignment. Intergroup comparison was carried out by one-way ANOVA with level of significance set at 5%.

**Results:**

The friction presented by the TRP group was statistically higher than that of the control group at 6 mm. It was also higher than in the control and CAP groups in terms of maximum friction.

**Conclusion:**

The customized acetate protector (CAP) demonstrated not to interfere in friction between the wire and the orthodontic bracket slot.

## Abstract (pt)

**Introdução:**

o aparelho ortodôntico fixo é considerado um fator causador de traumas na mucosa bucal. Com o intuito de controlar o desconforto no tecido mole, diversos ortodontistas recomendam a utilização de uma pequena quantidade de cera utilidade sobre os braquetes como forma de proteção. Esse estudo teve como objetivo avaliar,* in vitro*, o atrito gerado por dois tipos de protetores de braquetes (protetor de acetato e de resina - PPA e PRT) durante os estágios iniciais do tratamento ortodôntico.

**Métodos:**

o atrito gerado pelos protetores no fio ortodôntico foi avaliado em unidades de teste de modelos experimentais. Esses modelos foram ligados a uma máquina de ensaios mecânicos que simulava o alinhamento do canino superior. A comparação intergrupos foi realizada pela ANOVA, com nível de significância de 5%.

**Resultados:**

a fricção apresentada pelo grupo PRT foi estatisticamente maior do que a do grupo controle ao nível de 6mm. Para o atrito máximo, a média do grupo PRT foi estatisticamente maior do que a dos grupos controle e PPA.

**Conclusão:**

o protetor de acetato demonstrou não interferir no atrito entre o fio e a ranhura do braquete ortodôntico.

## INTRODUCTION

Fixed orthodontic appliances have been regarded as a common causative factor of oral ulcers and cellular alterations due to an intermittent friction between the oral mucosa and the orthodontic brackets.^1,2^ Such problem invariably leads to discomfort for both lingual and oral mucosa.^3^

To manage soft tissue discomfort commonly associated with fixed orthodontic appliances, most orthodontists recommend the use of a small amount of utility wax over the brackets in order to alleviate trauma. This is perhaps one of the most traditional ways of protecting soft tissues during orthodontic treatment. Although incorporation of anesthetic compounds has been reported,^1^ no further investigation has been published on this type of protection. Other materials such as temporary restorative resins^4^ (e.g. Fermit^TM^) or even plastic materials have recently been used to cover brackets; however, the literature still lacks data on biocompatibility and influence of these materials on biomechanics.

With regard to orthodontic biomechanics specifically, previous studies have shown that the continued use of protectors (plastic or resin based) can increase friction between the wire/ligatures and the orthodontic bracket.^5-13^ Ideally, it would be desirable that materials used over brackets did not interfere in or even touch the wire and the bracket slot during orthodontic tooth movement.

An acetate protector customized for each type of bracket, bearing the aforementioned concept of not interfering with either the wire or bracket slot, was developed for the present study. Such type of protector still needs to be validated through a randomized controlled trial (RCT) before its use can be fully recommended. However, it would be unethical to start an RCT without first checking whether this type of protector design could interfere in orthodontic movement. To date, it appears that no study has yet been published on the use of such device, or even on its influence in relation to friction development.

The aim of the present *in vitro* study was to compare the friction generated by two different groups of bracket protectors when simulating the leveling of a maxillary canine: (1) Brackets covered with a customized acetate protector, fabricated for the present study, and (2) Brackets covered with a temporary composite resin. Both groups were compared to a control group with no protection.

## MATERIAL AND METHODS

### Type of brackets

The preadjusted brackets (Roth prescription, Dental Morelli, São Paulo, Brazil) used in the present study were made of stainless steel 0.022-in slot, specifically designed for right upper premolars, canines and lateral incisors. Except for the lateral incisor bracket, all of them contained a hook.

### Experimental groups

Three groups were created in order to compare the degree of friction: CAP group, with brackets covered with a customized acetate protector (CAP); TRP group, with brackets covered with a temporary resin protector (TRP); and control group, with brackets without protection for soft tissues. Twenty-one friction tests were performed on each group. The number of tests was determined by a sample size calculation based on a pilot study with 10 tests, in which a difference of 1 N among the means was considered to be clinically significant. For this calculation, the power of the test was set at 80%, and the level of significance at 5%.

### Fabrication of the customized acetate protectors, as used in CAP group

The customized acetate protectors (CAP) used to cover metallic bracket surfaces (Dental Morelli, São Paulo, Brazil) were fabricated by a single calibrated operator. The calibration process included fabrication of a series of different protectors, and was determined upon the authors' satisfaction with the quality of the protectors produced. Sixty protectors were fabricated for each type of bracket used in the study. Direct contact of the acetate material with either the orthodontic wire or the bracket slot was avoided by relieving the slot of each bracket with light-activated Z100 composite resin (3M, St. Paul, MN, USA). Such relief is part of the protector fabrication process. It is something that needs to be done only on the brackets used for fabrication, not on the brackets where the protectors will be used. Clinically, the rationale is exactly the same, and the relief is hence unnecessary on the brackets used by the patient.

The brackets were then placed in a vacuum thermoforming machine so as to obtain a 1 mm-thick acetate pellicle. The acetate was cut with 0.5-mm steel discs which were set on a slow speed straight handpiece. The cutting procedure was performed in such a way so as to restrict the protector to the bracket surfaces that normally touch soft tissues. During this procedure, any areas or points of contact between the orthodontic wire and the acetate material were removed.

### Fabrication of temporary resin protectors, as used in the TRP group

Temporary resin protectors (TRP) consisted in the application of a standardized quantity of Fermit composite (Ivoclar Vivadent Inc., Amherst, NY, USA) on the center of the bracket by means of light digital pressure. This procedure simulated the clinical application of this material as a soft tissue protector for orthodontic patients.

In order to avoid excess, the amount of composite resin applied was standardized. Such standardization was obtained by dispensing a 2-mm long straight line of resin over a millimetric ruler. This amount was enough to protect each bracket. Any possible excess touching the wire outside the slot was removed with the aid of an explorer.

### Friction evaluation

The *in vitro* experimental model (test unit) used to assess friction was based on a previously described methodology,^14^ in which the right buccal segment of the upper arch containing five stainless steel 0.022-inch preadjusted brackets (from the second premolar through the central incisor) was reproduced.

One of the main differences between the model used and that already described^14^ is the height of the maxillary canine: 6 mm in the present study, and 3 mm in the previous publication.

Brackets were passively aligned with a 0.0215 x 0.028-in stainless steel wire (Dental Morelli, São Paulo, Brazil). The next step consisted in attaching them to the vertical metallic bars of the test unit by using superbond glue (Loctite Brand - Consumer Products, Henkel Corporation, Westlake, Ohio, USA). The gluing procedure was preferred to the mechanism described for the original prototype^14^ because of greater stability. The interbrackets distance was 6 mm. The only metallic bar allowed to move was the one containing the bracket for the maxillary canine. During the whole procedure, no accidental bracket debonding was observed.

**Figure 1:**
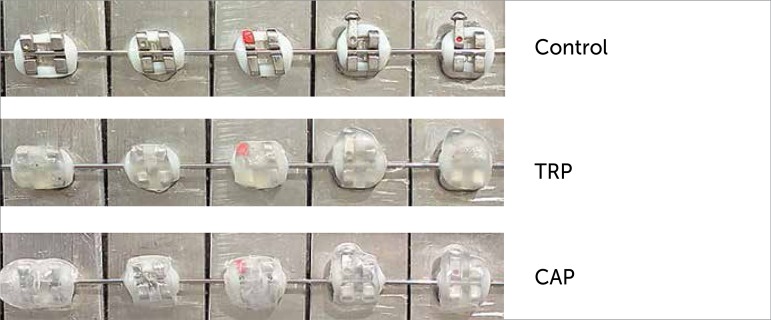
Two systems containing protected brackets (CAP - customized acetate protector and TRP - temporary resin protector), and one system without protected brackets (control).

The tests were performed with 0.014-in superelastic nickel titanium wires tied to the bracket slots by means of elastic ligature ties (Dental Morelli, São Paulo, Brazil). The model simulated a severely crowded maxillary canine. As the simulated hemiarch did not have a parabolic shape, the segments of wire used in this study were straight, and measured 55 mm in length. Every wire segment received standardized cuts, and was installed into the test unit with a 7.5 mm wire excess in both extremities of the hemiarch. The wire, ligature tie and bracket protectors (CAP and TRP) were replaced by new ones before the start of each test. The same experimental model and brackets were used throughout the study. Figure 1 illustrates each system separately (CAP, TRP and control).

**Figure 2:**
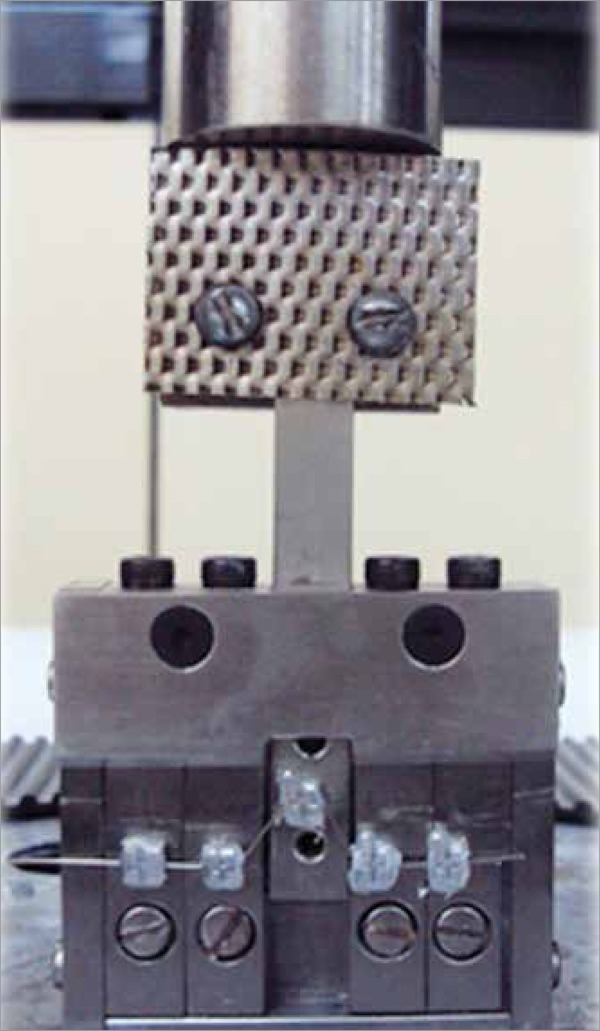
Tensile strength of 6 mm being performed on the vertical bar of the experimental model, by a mechanical testing machine.

In order to measure the friction produced in each test, the model was attached to a mechanical testing machine (Shimadzu, AG-1, 250kn, Tokyo, Japan) that had been previously calibrated by the manufacturer (Fig 2).

Instead of pulling the wire ends to align the maxillary crowded canine, it was preferred to elevate the mobile metallic bar from a position leveled 6 mm above. This proved to be useful to eliminate small random friction variations between the mobile and adjacent fixed metallic bars. The mobile metallic bar was elevated by the capture mechanism of the testing machine, using a 10 N load cell. This adaptation produced a mean friction value close to zero in several pre-tests performed without orthodontic wire at a speed of 6 mm/min. This value served as reference to adjust to zero the friction inherent to the experimental model before starting each test.

Maximum friction values and friction values at 6 mm of elevation of the maxillary canine bracket (static friction) were both calculated for all tests performed on each group. Tests were performed in dry conditions and room temperature at 25 ± 2ºC.

To eliminate a potential performance bias associated with bracket fatigue, the first sequence in which the groups were tested was randomly established. The following sequences were determined by a combinatorial analysis.

Data were collected and stored into SPSS (Statistical Package for the Social Sciences, SPSS Inc, Chicago, USA, version 10.0 for Microsoft Windows), and submitted to statistical analysis. The evaluation of sample distribution was carried out by the Shapiro-Wilk test at 5%, in order to detect normal distribution.

The means, standard deviation and confidence interval at 95% were obtained for maximum friction and for friction at 6 mm produced on each group. Intergroup comparison was carried out by one-way ANOVA with level of significance set at 5%. Bonferroni test with the same level of significance was used to determine potential statistical differences between paired groups.

## RESULTS

Descriptive analysis and intergroup comparison are available for friction at 6 mm (Table 1) and maximum friction (Table 2).

**Table 1:** Descriptive analysis and intergroup comparison for friction at 6 mm.

Group	n	Mean (Newtons)	Standard-deviation (Newtons)	Confidence interval at 95%
Control (6 mm)	21	7.67A	0.59	7.39 – 7.94
CAP (6 mm)	21	8.10A	0.64	7.79 – 8.37
TRP (6 mm)	21	9.90B	1.23	9.90 – 9.34

Different letters represent statistically significant difference between groups (P = 0.05).

**Table 2:** Descriptive analysis and intergroup comparison for maximum friction.

Group	n	Mean (Newtons)	Standard deviation (Newtons)	Confidence interval at 95%
Control (maximum friction)	21	7.97A	0.58	7,70 – 8,23
CAP (maximum friction)	21	8.29A	0.60	8,01 – 8,56
TRP (maximum friction)	21	10.20B	1.28	9,61 – 10,79

Different letters represent statistically significant difference between groups (P = 0.05).

For both types of friction, ANOVA detected statistically significant difference between groups (friction at 6 mm: P = 0.000; maximum friction: P = 0.000).

Paired group comparisons performed for friction at 6 mm (Table 1) resulted in no statistically significant difference between the control and CAP groups (P = 0.396). Friction presented by the TRP group was statistically higher than those of the control (P = 0.000) and CAP (P = 0.000) groups.

With regard to maximum friction (Table 2), no statistically significant difference was found between the control and the CAP group (P = 0.340). TRP friction was statistically higher than friction produced by the control (P = 0.000) and by the CAP (P = 0.000) group.

## DISCUSSION

For a 6-mm crowded maxillary canine, CAP data were not higher than data from the control group (unprotected brackets). This magnitude of canine crowding was regarded as severe in previous reports.^15^ Even under such circumstances, the fabricated customized acetate protectors produced a friction quite similar to that seen in conventional systems, in which no protection is employed.

We believe that such result was obtained because of careful protector fabrication in which any contact between the acetate, the wire and the slot was fully avoided. Another literature report published in 2009^10^ tested the friction level produced by an elastic ligature of which design also intended to avoid any type of contact with both the wire and the bracket slot. Similar to what we observed with our customized acetate protector, the authors also reported a lower friction degree of their elastic ligatures in comparison to conventional ligatures. Such tendency in reducing the friction degree when using either special ligatures^8,11,15,16^ or passive self-ligating brackets^17,18^ has been shown in the literature.

It is also important to bear in mind that contact between different materials collaborates to increase friction. A previous study^12^ tested a system in which all components were metallic: the wire, the ligature and the slot. This scenario yielded a decrease in friction similar to what is seen with self-ligating brackets. The acetate protectors used in the present study went through a very careful fabrication process focused on avoiding any contact between the protector and the other components of the system. This measure was particularly reinforced at the level of bracket slot entrances.

Because friction increases as wire thickness increases,^19^ it would have been interesting if rectangular wires had been tested as well. However, soft tissue protectors are mostly needed during the initial phases of orthodontic treatment (leveling and alignment). For this reason, a superelastic round wire seemed to be more appropriate to simulate the correction of a blocked maxillary canine. The chosen diameter of 0.014-in was based on a study^8^ in which the same type of malocclusion was simulated. Nevertheless, instead of calculating the mean of all frictions produced at several points along the 6 mm of correction, we decided to assess the greatest friction therein produced (maximum friction). Because of the large number of points along 6 mm of misalignment, and also due to the expected low values in Newton (N), the maximum friction appeared to be more representative of the obstacle to tooth movement.

In contrast to the methodology previously described,^14^ the 19 mm inter-bracket distance was reduced to 6 mm in order to be more in line with clinical reality. In the present study, only the wires and ligatures were changed in between tests, differently from previously published studies,^14^ in which brackets, wires and elastic ligatures were changed every five tests. We considered bracket replacement to be a disadvantage, since their replacement could increase the odds of standardization error. We also believe that the random order in which the groups were tested might have solved the bias related to bracket fatigue.

In addition to the improved level of comfort that patients are likely to experience with this customized acetate protector, the findings of the present study suggest that it does not interfere in orthodontic mechanics. As the tested protector does not seem to increase friction, it is believed that it may not interfere in treatment time, although treatment time does not seem to be solely dependent on friction, but also on the binding of the wire against the corners of the bracket.^6^ Nevertheless, we must bear in mind that such a problem is more common during sliding mechanics, not during leveling of canines as it is the case illustrated here.

When it comes to clinical feasibility, we do not expect clinicians to manufacture every single protector for their patients. Instead, we expect the orthodontic industry to manufacture kits of protectors that fit their different bracket designs. As long as the principles described here are incorporated into the industrialization process in a standardized manner, there shall be no concerns regarding technique sensitivity.

Although RCT studies conducted to evaluate the level of comfort of the acetate protectors are indeed necessary, the authors are confident in terms of material stability under the harsh conditions of the oral environment. The proposed acetate protectors can only increase friction if they shrink in contact with saliva. However, this seems to be highly unlikely as this is a material that is prone to absorb rather than lose components. On the other hand, virtually all composite materials undergo polymerization shrinkage, thereby increasing the chances of friction between the wire and the bracket slot.

In the future, it seems appropriate to carry out similar studies using thicker wires in order to simulate space closure mechanics. *In vitro* biosafety and temperature tests must also be conducted.

## CONCLUSION

It was possible to conclude that the use of an acetate bracket protector, such as proposed in the present *in vitro* study, results in a friction level similar to that observed with unprotected brackets. An opposite outcome was obtained when using a temporary resin as a form of protection to soft tissues.
